# 

**Published:** 1889-04

**Authors:** 


### CONTENTS.

### COMMUNICATIONS.

President's Address .......... 161
The Nervous Patient .......... 164
Calcific Inflammation .......... 167
Association .......... 170
Mistakes .......... 172
The Use of the Electro-Magnetic Mallet .......... 174
Short Notes on Diseases of the Palate .......... 178
Monthly Digest .......... 182

### PROCEEDINGS.

Ohio College of Dental Surgery .......... 192

### SELECTIONS.

The Nail Brush Question .......... 194
Method of making sections of teeth and preserving the delicate parts 196
To Preserve Blood Corpuscles for Microscopic Examination .......... 197
Absorption of the Tissues of the Mouth from Pressure .......... 197
Therapeutics of Iodol .......... 198
The Tooth in the Apple .......... 199
Influence of Certain Medicinal Agents on the bacillus of tubercle in man .......... 205

### EDITORIAL.

Libraries .......... 206
Ohio District Dental Societies .......... 208
American Medical Association .......... 209
Literary Items .......... 210
Bibliographical .......... 210
Biographical .......... 211

